# Simultaneous double dislocation of the interphalangeal joint of the same finger: a report of two cases

**DOI:** 10.11604/pamj.2014.19.400.5933

**Published:** 2014-12-30

**Authors:** Yasuhiro Seki

**Affiliations:** 1 Suwa Central Hospital, Department of Orthopaedic Surgery, Tamagawa, Chino-city, Japan

**Keywords:** Finger dislocation, manual reduction, early range of motion

## Abstract

Simultaneous dislocation of both the proximal and distal interphalangeal (PIP and DIP) joints in a finger is uncommon. Two patients were treated conservatively. Both two patients fell from a step-ladder and X-rays revealed dorsal dislocations of both PIP and DIP joints of their right little fingers. Manual reduction was easily achieved with gentle longitudinal traction. The mechanism of the injury is believed to be hyperextension of both the DIP and PIP joints. Closed reduction is the treatment of choice and early active range of motion to prevent joint contracture should be recommended.

## Introduction

Single dislocation of the finger is a common injury; however, simultaneous dislocation of both proximal and distal interphalangeal (PIP and DIP) joints in a finger is uncommon. We treated two cases conservatively.

## Patient and observation

**Figure 1:**
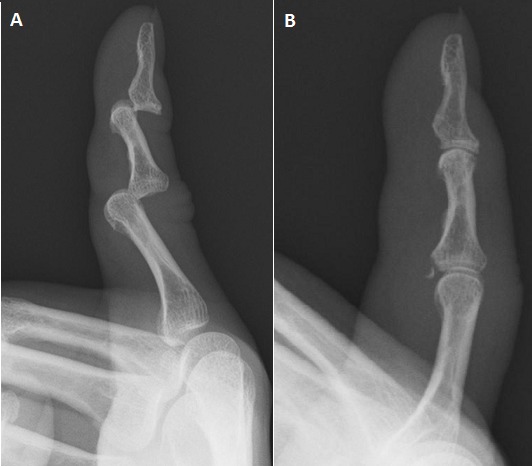
Case 1 (A) X-rays of the little finger showing dorsal dislocation of both the proximal and distal interphalangeal joints (step-ladder deformity); (B) Post-reduction X-rays confirming that both joints are well reduced, but the PIP joint had a volar rim fracture

**Case 1:** a 64-year-old man fell from a step-ladder, injuring the right little finger. On physical examination, the finger was found to be deformed, and an X-ray demonstrated a dorsal dislocation of both the PIP and DIP joints (Figure 1A). Manual reduction was easily achieved with gentle longitudinal traction; however, the X-ray after reduction (Figure 1B) revealed an avulsion fracture of the volar proximal rim of the middle phalanx. A dorsal splint was applied for three weeks and then rehabilitation was started. At the final review there was no joint instability, whereas the PIP flexion was 90 degrees and the extension deficit was 10. The range of motion had a slight limitation; however, the patient had no complaint of pain or functional deficit.

**Figure 2:**
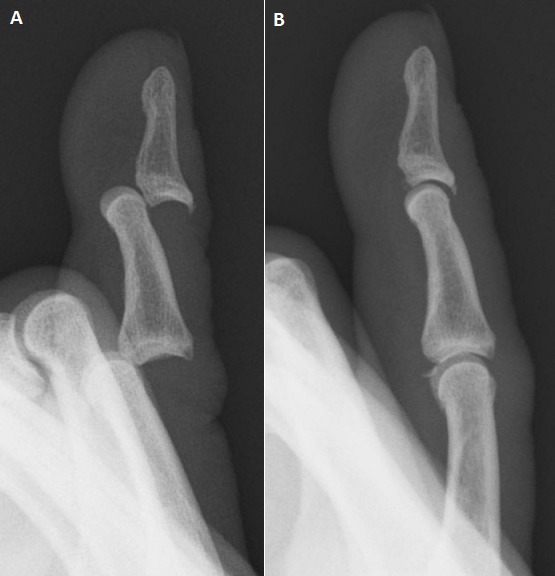
Case 2 (A) X-rays of the little finger showing dorsal dislocation of both interphalangeal joints; (B) Post-reduction X-rays with a volar rim fracture

**Case 2:** a 66-year-old man fell from a step-ladder and injured his right little finger. The X-ray revealed dorsal dislocation of both PIP and DIP joints (Figure 2A). Gentle longitudinal traction was successful, but the X-ray showed a volar rim fracture (Figure 2B). A splint was applied for one week. Since the patient did not complain of pain or joint instability a week after the injury, buddy taping was applied instead of the splint and nearly full range motion was achieved at the final review.

## Discussion

Simultaneous interphalangeal double dislocations are rare. The majority of dislocations of this type occurred during ball sports, but some cases were caused by a fall [1]. The mechanism of the injury is believed to be hyperextension of both the DIP and PIP joints. The position of the hand at the time of accident, along with the direction of force from volar to dorsalward determined the hyperextension initially of the distal joint. The forces are then not sufficiently dissipated, and a subsequent tear of the volar capsule of the proximal joint occurs, with hyperextension, causing the base of the middle phalanx to dislocate on the dorsum of the head of the proximal phalanx [2]. Therefore, strictly speaking, it is not a “ simultaneous” dislocation. Closed reduction is the treatment of choice and longitudinal traction is sufficient in most cases. Because immobilisation was applied for three weeks and slight contracture remained in case 1, we started finger motion exercise one week after the injury and obtained better range of motion in case 2. Mangelson [3] researched complications following a single dislocation of the PIP joint and recommended early-supervised active range of motion as permitted by stability to prevent stiffness and contracture. He suggested that even if there was collateral ligament injury or volar rim fracture (generally less than 30% of articular surface) of the middle phalanx, finger motion exercise had better begin immediately under a figure-of-eight splint or buddy taping. Early active range of motion to prevent joint contracture for not only a single PIP joint dislocation, but also double (both PIP and DIP joints) dislocations should be recommended.

## Conclusion

The mechanism of simultaneous interphalangeal double dislocations is believed to be hyperextension of both the distal and proximal interphalangeal joints. Closed reduction is the treatment of choice and longitudinal traction is mostly sufficient. Finger motion exercise should start as early as possible to prevent joint contracture under a figure-of-eight splint or buddy taping.
